# Sulfated Lewis A from the oviduct reservoir selectively binds to camel (*Camelus dromedarius*) sperm and extends their lifespan *in vitro*

**DOI:** 10.14202/vetworld.2025.1147-1155

**Published:** 2025-05-13

**Authors:** Mohamed M. M. El-Sokary, Hamad A. Albreiki, Salem Belal, Latifa R. Alshamsi

**Affiliations:** 1 Higher Colleges of Technology, Faculty of Health Science, Abu Dhabi, 17155, UAE; 2 Department of Theriogenology, Faculty of Veterinary Medicine, Benha University, Benha, 13511, Egypt

**Keywords:** camel, glycan binding, oviduct, reproductive biotechnology, sperm viability, sperm, sulfated Lewis A

## Abstract

**Background and Aim:**

Camel reproduction faces significant challenges, including poor semen preservation and a limited understanding of gamete interactions, particularly within the oviduct. Glycan-mediated sperm binding in the oviduct is pivotal for sperm storage and longevity in various species. This study aimed to evaluate the binding affinity of camel epididymal sperm to sulfated Lewis A (SuLeA) – a trisaccharide from the oviductal isthmus – and investigate its effect on sperm lifespan and viability *in vitro*.

**Materials and Methods:**

Fluorescent-labeled SuLeA was used to localize glycan-binding sites on camel sperm. An *in vitro* model involving biotinylated SuLeA conjugated to streptavidin-sepharose beads was developed to mimic oviductal interactions. Sperm-oviduct binding specificity was assessed by pre-incubating sperm with SuLeA before their exposure to epithelial cell aggregates. Sperm viability was evaluated over 48 h using SYBR-14 and propidium iodide staining.

**Results:**

Fluorescent SuLeA showed preferential binding to the post-acrosomal region of camel sperm (53%, p < 0.05). Pre-incubation with SuLeA significantly inhibited sperm adhesion to oviductal aggregates (82% vs. 25%, p < 0.05), confirming binding specificity. Sperm demonstrated a high affinity to immobilized SuLeA (5 sperm/bead), which was reduced to 1 sperm/bead following glycan pre-incubation. Notably, sperm bound to immobilized SuLeA exhibited significantly higher viability (59%) after 48 h compared to unbound sperm (5%, p < 0.05).

**Conclusion:**

This study establishes that SuLeA selectively binds to camel sperm at the post-acrosomal region, mimicking physiological sperm-oviduct adhesion. The interaction not only confirms glycan specificity but also significantly prolongs sperm viability. These findings provide a promising foundation for developing freeze-free preservation techniques and improving artificial insemination protocols in camelids.

## INTRODUCTION

In the Middle East, camels (*Camelus dromedarius*) play a vital role in cultural heritage and economic sustainability, contributing to the production of milk and meat, and participating in recreational activities such as racing and competitive exhibitions [1]. Despite their significance, reproductive challenges continue to hinder this species, with fertility problems substantially limiting reproductive efficiency in both male and female camels. Low conception rates and male infertility are commonly reported issues [2]. One of the primary obstacles to advancing reproductive management in camels is the insufficient understanding of the physiological mechanisms underlying fertility, particularly gamete interactions and embryonic development. Furthermore, semen preservation presents a considerable challenge, as sustaining its viability and fertilizing potential after cryopreservation is crucial for the success of artificial insemination programs. These constraints have impeded the broader application of assisted reproductive technologies (ARTs) in camels [3]. Despite progress in semen cryopreservation techniques, dromedary camel semen continues to exhibit low post-thaw viability and fertilizing capability, thereby limiting its practical use [4]. In light of these challenges, enhancing sperm preservation strategies has garnered growing interest, especially with the increasing demand for artificial insemination and the conservation of valuable genetic resources [5]. Among the novel approaches under investigation, glycan-mediated interactions have emerged as a promising avenue for supporting sperm viability and functionality during storage.

Following ejaculation, sperm are rapidly transported to the oviduct through rhythmic contractions of the myometrium. The duration of this transit varies depending on the species and the site of semen deposition. For instance, in cattle, sperm can reach the ampulla within minutes; however, many accumulate and remain in the isthmus for several hours, with substantial concentrations observed approximately 8 h after ejaculation [6]. In contrast, in horses, this process occurs within 2–4 h [7]. The mechanisms responsible for sperm retention within the oviductal isthmus have been well-documented, with evidence indicating that the physical attachment of spermatozoa to the oviductal epithelial cells (OEC) constitutes the primary mode of storage [8]. This binding is facilitated by interactions between the sperm plasma membrane and the mucosal epithelium of the oviduct. Lefebvre *et al*. [8] have emphasized the role of glycan-binding proteins located on the sperm surface, which recognize specific glycans on the oviductal epithelium, leading to protein-carbohydrate interactions that establish and sustain the sperm reservoir [8]. Among these glycans, fucose-containing structures – especially the trisaccharide Lewis A – have been identified as key mediators of sperm-oviduct adhesion [9]. Research on the bovine sperm reservoir has demonstrated that sulfated Lewis A (SuLeA), a trisaccharide synthesized by the oviduct, features terminal fucose linked in an α1-4 configuration to N-acetylglucosamine. Moreover, sulfation may contribute to preserving sperm surface characteristics and preventing aggregation during storage. It may also protect Lewis A from enzymatic degradation, thereby maintaining its functional integrity. In addition, SuLeA exhibits specificity and saturable binding characteristics [10]. High-quality sperm adhere to the oviductal epithelium and persist in a quiescent state with reduced motility and suppressed capacitation [11].

Despite the crucial reproductive and economic role of dromedary camels in arid and semi-arid regions, their fertility remains suboptimal due to limited advancements in ART. One major limitation is the inadequate preservation of semen viability and functionality during storage, particularly under freeze-free conditions. While studies in other livestock species have demonstrated that glycan-mediated sperm-oviduct interactions are essential for sperm storage and longevity, there is a paucity of data on the molecular basis of these interactions in camels. Specifically, the role of SuLeA trisaccharide – an oviduct-derived glycan known to support sperm binding and survival in bovines – has not been elucidated in camelids. The absence of functional models replicating the camel oviductal microenvironment further constrains the development of effective sperm preservation strategies tailored to this species.

This study aimed to investigate the interaction between SuLeA and camel epididymal sperm, focusing on its binding specificity, localization on the sperm membrane, and its impact on sperm viability during *in vitro* incubation. By developing an immobilized glycan model, the research seeks to mimic the physiological binding mechanism of sperm to the oviductal epithelium, thereby providing insights into the potential of SuLeA as a biomimetic agent for enhancing sperm longevity. The ultimate objective is to inform the development of novel, freeze-free sperm preservation techniques that may improve the efficacy of ART in camel reproduction.

## MATERIALS AND METHODS

### Ethical approval

This study was conducted in accordance with ethical guidelines and was approved by the Research Ethics and Integrity Committee at the Higher Colleges of Technology under approval number REIC2024-FAC35. All experimental procedures complied with institutional and international standards for the ethical use of biological materials in research.

### Study period and location

The study was conducted from September to November 2024. The samples for this study were collected from a local slaughterhouse in the Al Ain region, Abu Dhabi.

### Chemicals

Unless otherwise stated, all chemicals were purchased from Sigma-Aldrich Co. (St. Louis, MO, USA). Biotinylated glycans were obtained from GlycoNZ (New Zealand). Sodium pyruvate (110.04), HEPES (M-15688), and Triton X-100 (686) were procured from Thermo Fisher Scientific, USA.

### Animals and sample collection

Testes with attached epididymides were collected from 31 adult dromedary camels (*C. dromedarius*), aged 5–10 years, at a local abattoir in the Al Ain region. A total of 62 testes were harvested in 10 separate batches, each containing samples from 3–4 animals. Immediately after slaughter, the samples were aseptically excised, immersed in phosphate-buffered saline (PBS) supplemented with 100 IU/mL penicillin and 100 μg/mL streptomycin, and transported in a temperature-controlled container at 4°C to the laboratory within 1.5 h to prevent post-mortem degradation.

### Epididymal semen processing and preparation

Upon arrival, testes and epididymides were rinsed in chilled (4°C) 0.9% physiological saline. The epididymides were dissected under sterile conditions, rinsed again in saline to remove residual blood, and surface sterilized by immersion in 70% ethanol. Semen was extracted by incision and pooled per batch. Aliquots (100 μL) were prepared and stored for downstream assays following the protocol described by Rashad *et al*. [12].

### Experimental design

#### Experiment 1: Labeling of sperm with fluorescently SuLeA

This experiment aimed to identify specific glycan-binding sites on the camel epididymal sperm membrane by labeling sperm with fluorescein-conjugated SuLeA glycan. The labeling process facilitated the visualization of binding patterns on the sperm surface using fluorescence microscopy. For each replicate, a minimum of 100 sperm cells was examined.

#### Experiment 2: Pre-treatment of oviduct epithelial cells with SuLeA

To evaluate whether pre-incubation with SuLeA affects sperm binding to camel OECs, sperm samples were first incubated with the glycan and subsequently co-incubated with oviductal cell aggregates. The number of sperm bound to the epithelial surface was quantified microscopically. Each experimental condition utilized 30 oviductal aggregates, and the experiment was independently replicated 3 times to ensure reproducibility. This cell-based binding assay was conducted *in vitro*.

#### Experiment 3: Binding of biotinylated SuLeA glycan to streptavidin-sepharose

In this biochemical assay, biotinylated SuLeA was immobilized onto streptavidin-sepharose beads to produce a glycan-coated matrix for subsequent functional interaction studies. A 10 μL bead suspension was used per reaction to prepare the glycan substrate. This approach enabled the controlled display of glycan motifs in a defined format, facilitating their interaction with sperm surface molecules in downstream assays.

#### Experiment 4: Viability assay of sperm bound to immobilized glycan

This experiment assessed the viability and persistence of sperm bound to immobilized SuLeA glycan over extended incubation periods. Sperm–bead co-incubations were performed and monitored at various time points ranging from 0.5 h to 48 h. Sperm viability was evaluated using SYBR-14 and propidium iodide (PI) staining under fluorescence microscopy. At each time point, a minimum of 100 beads per droplet was examined to determine the proportion of live and dead sperm, thereby assessing the extent to which glycan interaction contributes to sperm longevity.

### Labeling of sperm with fluorescently SuLeA

**Figure 1:**
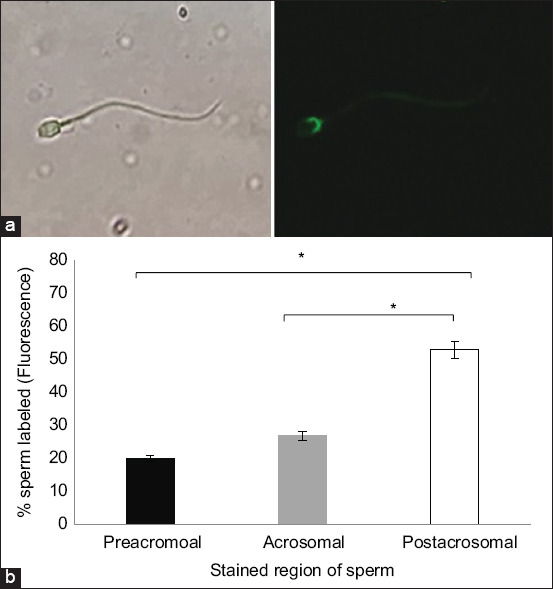
Localization of sulfate Lewis A on epididymal camel sperm. (a) Fluorescence microscopy shows sulfated Lewis A (SuLeA) binding to sperm. The left panel displays fluorescence (green), indicating binding in the post-acrosomal region, whereas the right panel shows the merged image with a bright-field microscopy scale bar = 50 μm. (b) SuLeA binding across three sperm regions: Pre-acrosomal, acrosomal, and post-acrosomal. The bars represent the percentage of sperm exhibiting fluorescence in each region. Asterisks indicate a significant difference (p < 0.05).

Fluorescent labeling of sperm with SuLeA glycan was conducted to identify glycan-binding sites (Figure 1a). The sperm concentration was standardized at 2 × 10^7^ cells/mL. Sperm were prepared in Tyrode’s Albumin Lactate Pyruvate medium (TALP) medium composed of 2.1 mM CaCl^2^, 3.1 mM KCl, 1.5 mM MgCl^2^, 100 mM NaCl, 0.29 mM KH^2^PO^4^ 0.36% lactic acid, 26 mM NaHCO_3_, 0.6% BSA, 1 mM pyruvic acid, 20 mM HEPES (pH 7.3), 10 U/mL penicillin, and 10 μg/mL streptomycin. Sperm were immobilized using 0.01% formaldehyde, ensuring that the membrane remained intact. Glycans conjugated to a 30-kDa fluorescein polyacrylamide carrier were used for labeling, with 0.8–0.9 μmol of glycan coupled per milligram of polyacrylamide. Fluorescein-labeled glycan was added to the sperm suspension at a final concentration of 50 μg/mL and incubated for 30 min at 39°C. After incubation, 10 μL of the sperm suspension was placed onto a microscope slide, covered, and examined under a fluorescent microscope. At least 100 sperm were assessed in each replicate [13].

### Retrieval of epithelial cells from the oviduct

Oviducts were obtained from apparently healthy female camels aged between 3.5 and 8 years, slaughtered at a local abattoir. Immediately after collection, the oviducts were placed on ice to preserve their cellular integrity and transported under cold conditions to the laboratory. Upon arrival at the laboratory, the OECs were carefully isolated and processed according to the method described by Winters *et al*. [14], with slight modifications to optimize cell recovery. The isolation procedure began with a thorough washing of the oviducts to remove any residual blood or debris. The epithelial sheet was then stripped from the washed isthmus using sterile techniques and resuspended in PBS. To separate intact epithelial cells, the suspension was centrifuged at 84× *g* for 1 min, ensuring gentle handling to maintain cell viability.

The resulting pellet was disaggregated by pipetting the suspension up and down 10 times with a 100 μL pipette to break apart any remaining cell clusters. Following this step, fresh PBS was added to the suspension, and the sample was centrifuged once more under the same conditions to further purify the epithelial cells. After isolation, the recovered cells were resuspended in a TALP medium to support cellular viability and functionality. The cell suspension was then evenly distributed into three separate 100-mm tissue culture dishes and incubated at 39°C for 90 min, allowing the cells to re-aggregate into organized structures. For subsequent sperm-binding experiments, only well-formed, spherical epithelial cell aggregates with diameters ranging between 100 and 150 μm were selected under a microscope [10]. These aggregates were then utilized to assess sperm-oviduct interactions, providing a valuable *in vitro* model for investigating sperm binding dynamics and cellular communication within the oviductal environment.

### Assay of sperm binding to oviduct epithelial cells pre-treated with SuLeA

**Figure 2:**
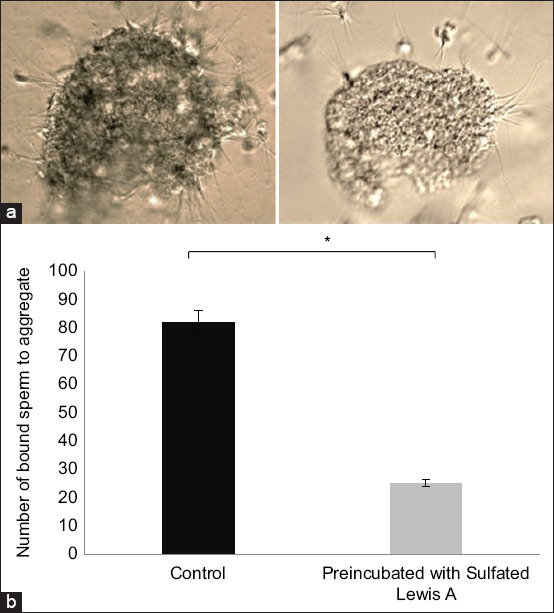
Inhibition of camel sperm from binding to oviductal aggregates by sulfated Lewis A (SuLeA). Camel sperm bind to oviductal aggregates isolated from the isthmus region. (a) In Panel A, the photo to the left shows many sperm attachments in the control group, whereas the photo to the right demonstrates a significant reduction in sperm binding following pre-incubation with SuLeA. Scale bar: 50 μm. (b) Panel B shows the number of sperm bound to oviductal aggregates before and after pre-incubation with SuLeA. Data are expressed as the mean ± standard error of the mean. Asterisks indicate statistically significant differences (p < 0.05).

As described by Winters *et al*. [14], with modifications implemented by El-Sokary *et al*. [15], the experimental procedure involved combining 30 oviduct cell explants with 20 μL sperm droplets, resulting in a final concentration of 1.6 × 10^6^ cells/mL. Before their introduction to the oviduct cell aggregates, sperm underwent a 30-min pre-incubation with SuLeA glycan. This step was essential for assessing the binding affinity of SuLeA to sperm lectins and determining whether it could effectively block these lectins, thereby potentially inhibiting sperm binding to the oviduct. Following incubation, any unattached or loosely bound spermatozoa were meticulously removed from the oviduct cell explants by careful washing with a TALP medium. This ensured that only firmly bound sperm remained associated with the oviduct aggregates. Subsequently, each aggregate was gently transferred onto a microscope slide, overlaid with a drop of TALP medium, and covered with a coverslip. The number of spermatozoa bound to the periphery of each aggregate was then quantified using phase-contrast microscopy under 400× magnification (Figure 2a).

### Binding of biotinylated SuLeA glycan to streptavidin-sepharose

**Figure 3:**
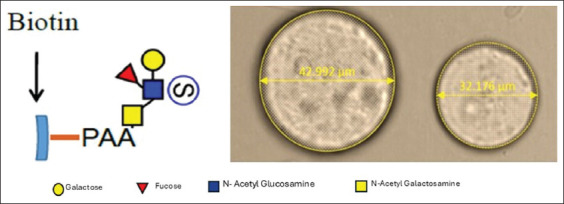
Characterization of biotinylated sulfated Lewis A (SuLeA) and its conjugation to sepharose beads. The left panel illustrates the structural components of biotinylated SuLeA, highlighting its sulfated trisaccharide moiety and biotin tag. The right panel presents sepharose beads of varying diameters (yellow), which serve as a solid-phase matrix for immobilization. These beads provide a controlled platform for studying sperm–glycan interactions, enabling the assessment of binding specificity and functional responses (scale bar 50 μm).

To prepare the affinity matrix, 10 μL of streptavidin-sepharose beads with an average particle size of 34 μm (Figure 3) were transferred to a 0.5-mL microcentrifuge tube and subjected to three sequential washes with 1× PBS (20 mM sodium phosphate, 0.15 M NaCl, pH 7.5). Each wash was carried out by centrifugation at 600 × *g* for 1 min, followed by careful removal of the supernatant and resuspension in fresh PBS. This step ensured the removal of any residual storage buffer or unbound components. The binding capacity of the streptavidin-sepharose matrix exceeds 300 nM of biotin per mL of suspension, corresponding to 6 mg of biotinylated BSA per mL. For glycan immobilization, 60 μg of biotinylated SuLeA glycan–polyacrylamide (glycan-PAA) was added to the washed beads in a total reaction volume of 20 μL [16].

The glycan binding reaction was carried out at room temperature (25°C) for 30–60 min with intermittent manual mixing. Tubes were gently flicked every 5 min to facilitate uniform glycan–sepharose interactions. Following incubation, unbound glycans were removed by washing the sepharose–glycan complexes 3 times with PBS, using the same centrifugation and resuspension protocol. In the final step, the pellet was resuspended in dmTALP to a final volume of 20 μL. The biotinylated glycan-PAA used in this study was a 30-kDa polyacrylamide conjugate with a molar composition of 20% SuLeA glycan and 5% biotin. As a control, a separate group of streptavidin–sepharose beads underwent the same washing and incubation procedure without the addition of biotinylated glycan to confirm the specificity of the glycan–sepharose interaction (Figure 3).

### Viability assay of sperm bound to immobilized glycan

**Figure 4:**
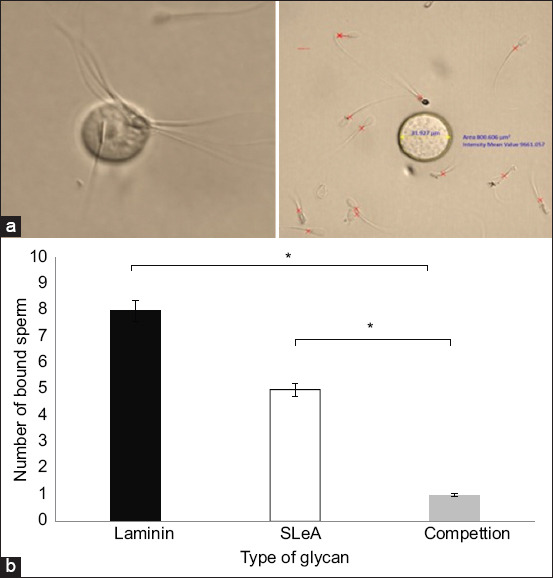
Competitive binding of camel sperm to immobilized sulfated Lewis A (SuLeA). Sperm are tested for their ability to bind to immobilized SuLeA (Panel A) (a), the image to the left shows sperm incubated directly with SuLeA-coated streptavidin beads, demonstrating specific binding. The right panel shows sperm pre-incubate with SuLeA, resulting in reduced attachment to the SuLeA-coated beads (scale bar = 50 μm). (b) The graph in Panel B illustrates the number of sperm attachments to laminin-coated and SuLeA-coated beads and the number of sperm after pre-incubation with SuLeA. Data are presented as values are presented as the mean ± standard error of the mean. Asterisks refer to a statistically significant difference (p < 0.05).

For sperm–bead interaction studies, semen samples were resuspended in 1 mL of dmTALP medium. Motility was evaluated immediately using phase-contrast microscopy. To determine sperm concentration, a 1:100 dilution was prepared by mixing 10 μL of sperm suspension with 990 μL of nanopure water. The sample was gently pipetted to ensure homogeneity. A 20-μL aliquot of this dilution was then loaded into each chamber of a hemocytometer. Sperm counts were conducted in either five central squares or all 25 squares, with a minimum threshold of 30 sperm to ensure accuracy. The concentration was adjusted to 800,000 sperm/300 μL. Aliquots were dispensed as 300-μL droplets into a 60-mm culture dish and overlaid with mineral oil to prevent evaporation (Figure 4a).

**Figure 5:**
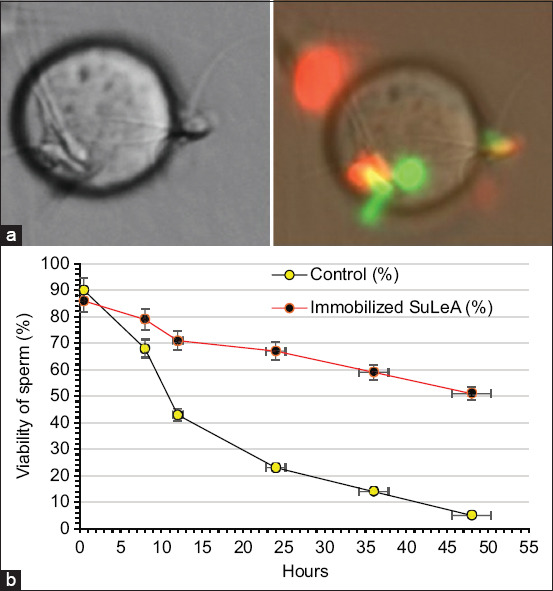
Sperm viability follows binding to sulfated Lewis A (SuLeA)-coated beads. (a) Panel A presents sperm bound to SuLeA (left) and fluorescence microscopy images of sperm bound to SuLeA-coated beads (left), with viability assessed using SYBR14 (green, live) and PI (red, dead) staining (scale bar = 50 μm). (b) Panel B illustrates sperm viability over time (up to 48 h) in comparison with free-swimming sperm.

Glycan–bead interactions were initiated by adding 10 μL of biotinylated SuLeA glycan-coated bead suspension into each sperm-containing droplet using a micropipette carefully inserted through the mineral oil layer [10]. Sperm–bead suspensions were incubated at 39°C in a gas environment containing 5% CO^2^ and 95% air. Incubation time points were 0.5, 8, 12, 24, 36, and 48 h to monitor sperm–glycan interactions over time. Following incubation, 100 nM SYBR-14 was added to stain live sperm, and 12 μM PI was used to mark non-viable sperm (Figure 5a). The number of sperm bound to each glycan bead was assessed using fluorescence microscopy at 400× magnification. For accurate quantification, a minimum of 100 beads per droplet was evaluated. If necessary, 4% paraformaldehyde was added to immobilize sperm for counting. Representative images of bound sperm were captured using a digital fluorescence microscope camera.

### Statistical analysis

Data were analyzed by analysis of variance using IBM SPSS version 22 (IBM Corp., NY, USA). The normality of the data was assessed before analysis. Tukey’s multiple comparison test was used to identify significant differences between means. Results are presented as mean ± standard error of the mean (SEM). Differences were considered statistically significant at p < 0.05.

## RESULTS

### Localization of SuLeA binding sites on camel sperm

The results demonstrated that a majority of camel sperm (53%) exhibited fluorescence in the post-acrosomal region, followed by the acrosomal (27%) and pre-acrosomal (20%) regions. Statistical analysis confirmed that fluorescence intensity in the post-acrosomal region was significantly higher than in both the acrosomal and pre-acrosomal regions (p < 0.05). These findings indicate a preferential interaction between SuLeA and molecular components localized within the post-acrosomal domain of the sperm membrane (Figure 1b).

### Pre-incubation of camel sperm with SuLeA reduces binding to oviduct explants

To assess the effect of SuLeA on sperm-oviduct adhesion, camel spermatozoa were incubated with oviductal explants obtained from the isthmus. In the control group, 82% of sperm bound to the epithelial surface, demonstrating strong adhesive capacity. However, sperm pre-incubated with SuLeA showed a significant reduction in binding, with only 25% of sperm adhering to the explants (p < 0.05). These results suggest that SuLeA competes with endogenous oviductal glycans for sperm surface receptors, thereby disrupting sperm-epithelium interactions (Figure 2b).

### Camel sperm bind to immobilized biotinylated SuLeA

To evaluate the specificity of sperm binding to immobilized SuLeA, biotinylated SuLeA was conjugated to Sepharose beads to provide a defined adhesion platform. Sperm incubated with laminin-coated beads exhibited an average of eight sperm per bead. In contrast, an average of five sperm per bead was observed for those incubated with SuLeA-coated beads. Notably, when sperm were pre-incubated with free SuLeA before exposure to the coated beads, binding was significantly diminished to approximately one sperm per bead (p < 0.05). These findings further confirm the specificity of camel sperm interactions with SuLeA and suggest that pre-incubation saturates available binding sites, preventing subsequent adhesion (Figure 4b).

### Effect of binding to immobilized SuLeA on sperm viability

The impact of sperm binding to immobilized SuLeA on sperm viability was examined at multiple time intervals: 0.5, 8, 12, 24, 36, and 48 h. Viability was assessed using dual staining with SYBR-14 and PI. Free-swimming sperm served as controls. At 0.5 h, sperm viability was approximately 90% and remained relatively stable at 86% after 8 h. A gradual decline was observed at later time points: 79% at 12 h, 71% at 24 h, 67% at 36 h, and 59% at 48 h. In contrast, free-swimming sperm exhibited a steep decline in viability, reaching only 5% by 48 h (p < 0.05). These results demonstrate that binding to immobilized SuLeA significantly preserves sperm viability over extended periods (Figure 5b).

## DISCUSSION

The ability of sperm to bind and interact with the oviductal epithelium is a fundamental mechanism in reproductive biology, functioning as a natural reservoir that prolongs sperm lifespan and preserves fertilization potential. In several mammalian species, this interaction is mediated by specific carbohydrate ligands present on the oviductal surface, which selectively bind to complementary receptors on spermatozoa [8]. The present study investigated the role of immobilized SuLeA trisaccharide in mimicking this oviductal binding mechanism in camels, thereby offering new insights into sperm preservation outside the reproductive tract. Understanding this interaction is vital for the development of advanced reproductive technologies aimed at improving artificial insemination success rates, optimizing semen storage protocols, and enhancing camel breeding programs.

This study revealed that camel sperm specifically bind to SuLeA, highlighting a targeted molecular interaction that may contribute to sperm-oviduct adhesion and reservoir formation. The data demonstrated preferential localization of SuLeA binding in the post-acrosomal region of epididymal sperm. The significantly higher binding observed in this region, compared to the acrosomal and pre-acrosomal areas (p < 0.05), suggests that SuLeA may interact with glycoproteins or receptors uniquely expressed in the post-acrosomal membrane. In contrast, a study by Dutta *et al*. [10] on bovine and swine sperm has shown glycan affinity localized to the apical ridge of the acrosome. The relatively lower fluorescence intensity in the acrosomal and pre-acrosomal regions in the present study could reflect differences in receptor distribution or accessibility, implying that specific sperm membrane proteins play active roles in oviductal adhesion. In addition, the absence of certain surface receptors – such as bovine seminal plasma (BSP)-A1/A2, which are present in ejaculated bovine sperm and exhibit binding affinity toward Lewis A in the acrosomal region – may explain the reduced binding observed in camel epididymal sperm [17].

This study also confirmed that camel sperm bind to oviductal cell aggregates derived from the isthmus, suggesting a functional role in reservoir formation. To validate the specificity of this interaction, a competitive binding assay was conducted by pre-incubating sperm with SuLeA. This treatment significantly reduced, though did not completely inhibit, sperm binding to oviduct aggregates. The partial inhibition may be attributed to additional glycans and carbohydrate ligands expressed in the oviductal epithelium, as well as the presence of sperm surface proteins such as BSP2 and BSP3, which are known mediators of sperm adhesion. These additional molecular interactions likely contribute to the establishment of the sperm reservoir alongside SuLeA [18].

To further investigate the functional role of SuLeA in sperm binding, we employed a molecular mimicry approach using biotinylated SuLeA conjugated to streptavidin-coated beads. This *in vitro* model system simulated the oviductal epithelial surface and enabled the assessment of glycan-mediated sperm interactions. Camel sperm incubated with SuLeA-functionalized beads exhibited a strong binding affinity, indicating a specific interaction with the glycan ligand. Pre-incubation of sperm with free SuLeA before exposure to the coated beads resulted in complete inhibition of binding, thereby confirming the specificity of the interaction. These findings align with previous studies by Machado *et al*. [19] and Sharif *et al*. [20] that used glycan-based models to investigate sperm-oviduct adhesion mechanisms.

The extended viability of camel sperm bound to immobilized SuLeA over prolonged incubation periods underscores the functional significance of this trisaccharide in preserving sperm integrity. In contrast to the rapid decline in viability observed in free-swimming sperm, the substantially higher survival rates of bound sperm suggest that the SuLeA-coated bead model creates a stabilizing microenvironment that mitigates the detrimental effects of prolonged exposure. The prolonged lifespan of bound sperm may be attributed to a quiescent state, characterized by reduced motility [21]. Similar observations in other species have shown that sperm bound to OECs exhibit lower intracellular Ca^²+^ concentrations, a mechanism believed to delay capacitation and preserve viability [22]. These findings are consistent with reports in other mammals, where binding to immobilized glycans prolongs sperm survival [10]. Although a gradual decline in viability was observed over time – expected due to ongoing metabolic activity – the delayed deterioration suggests that immobilized SuLeA may help attenuate oxidative stress or apoptosis-related pathways that contribute to sperm degradation [23].

## CONCLUSION

This study provides novel insights into the role of SuLeA glycan in mediating sperm-oviduct interactions and preserving sperm viability in dromedary camels (*C. dromedarius*). The results demonstrate that SuLeA binds preferentially to the post-acrosomal region of epididymal sperm, indicating the presence of specific receptors that mediate this interaction. Competitive inhibition assays confirmed the specificity of this glycan-sperm binding, as pre-incubation with free SuLeA significantly reduced sperm adhesion to both OECs and SuLeA-functionalized beads (p < 0.05). Furthermore, sperm bound to immobilized SuLeA exhibited significantly prolonged viability over 48 h compared to unbound, free-swimming sperm, highlighting its potential role in creating a quiescent and protective microenvironment similar to the *in vivo* oviductal reservoir.

A major strength of this study lies in the integration of a biomimetic model using immobilized SuLeA to simulate the oviductal environment. This approach enabled the dissection of specific glycan-mediated interactions while assessing functional outcomes such as sperm adhesion and viability. The use of dual staining (SYBR-14 and PI) for viability assessment and time-course analysis provided robust data on the protective effects of SuLeA binding over time.

However, this study is not without limitations. The use of epididymal rather than ejaculated sperm may not fully capture the complete repertoire of surface receptors involved in post-ejaculatory binding dynamics. In addition, while SuLeA was shown to play a key role, the partial inhibition of sperm-oviduct adhesion suggests the involvement of additional glycans or protein mediators that warrant further exploration.

Future studies should focus on characterizing the full spectrum of glycan receptors on ejaculated camel sperm and elucidating the cooperative role of multiple oviductal ligands. Investigating the intracellular signaling pathways activated by glycan binding could further clarify mechanisms that underlie sperm longevity and capacitation delay. Moreover, extending this biomimetic approach to develop freeze-free sperm preservation media or coatings could significantly enhance the efficiency of artificial insemination protocols in camels and other livestock species.

## DATA AVAILABILITY

The data that support the findings are available from the corresponding author upon a request.

## AUTHORS’ CONTRIBUTIONS

MMME: Conceptualized, designed and performed the experiments, collected the data, performed critical revisions, and drafted and reviewed the manuscript. HAA, SB, and LRA: Performed the experiments, collected data, and drafted the manuscript. All authors have read and approved the final manuscript.
